# High Mobility Group A 1 Expression as a Poor Prognostic Marker Associated with Tumor Invasiveness in Gastric Cancer

**DOI:** 10.3390/life12050709

**Published:** 2022-05-09

**Authors:** Hung-Pin Chang, Jen-Tang Sun, Chiao-Yin Cheng, Yao-Jen Liang, Yen-Lin Chen

**Affiliations:** 1 Department of Emergency Medicine, Far Eastern Memorial Hospital, New Taipei City 220, Taiwan; 92825@mail.femh.org.tw (H.-P.C.); tangtang05231980@mail.femh.org.tw (J.-T.S.); 410068068@mail.fju.edu.tw (C.-Y.C.); 2 Graduate Institute of Applied Science and Engineering, Fu-Jen Catholic University, New Taipei City 242, Taiwan; 071558@mail.fju.edu.tw; 3 Department of Pathology, Tri-Service General Hospital, National Defence Medical Center, No. 325, Sec. 2, Chenggong Rd., Neihu District, Taipei 114, Taiwan

**Keywords:** high mobility group A 1 (HMGA1), gastric cancer, proliferation, epithelial-mesenchymal transition

## Abstract

The prognosis of advanced gastric cancer remains poor. Overexpression of high mobility group A 1 (HMGA1) in breast cancer and neuroblastoma indicates a poor prognosis. However, the relationship between HMGA1 expression and gastric cancer development remains unclear. Treatment strategies can be developed by identifying potential markers associated with gastric cancer. We used a constructed tissue array and performed hematoxylin and eosin and immunohistochemical staining. We quantified the staining results and performed statistical analysis to evaluate the relationship between HMGA1 expression and prognosis. HMGA1 expression was related to the expression of Ki-67, caspase3, CD31, N-cadherin, fibronectin, pAkt, and pErk. In the Kaplan–Meier graph, higher HMGA1 expression levels were associated with a relatively poor survival rate (*p* = 0.04). High expression of HMGA1 leads to a low survival rate, which is associated with HMGA1, proliferation, apoptosis, angiogenesis, epithelial-mesenchymal transition, and tyrosine kinase.

## 1. Introduction

Gastric cancer is a serious disease associated with a poor prognosis. Globally, more than one million people are diagnosed with gastric cancer annually. Moreover, this cancer ranks among the top ten causes of cancer-related deaths [1,2,3]. With advances in medical technology, radiotherapy, and chemotherapy, the 5-year survival rate of early gastric cancer has reached >90–95% [4,5,6], whereas late-stage gastric cancer has a much poorer prognosis. Therefore, biomarkers with prognostic value for gastric cancer are urgently needed. Abnormal expression of many genes has been proposed in gastric cancer, such as HER2, EGFR, ERBB-2, MET, and TP53 (tumor protein p53) [7]. *Helicobacter pylori* infection also causes gastric cancer. These patients show reduced expression of the microRNA Let7, which directly regulates high mobility group A (HMGA) 2 [8]. However, HMGA1 is not specifically expressed in gastric cancer.

HMGA protein family consists of four members, divided into the major categories of HMGA1 and HMGA2. HMGA1 can be divided into HMGA1a, HMGA1b, and HMGA1c, whereas HMGA2 has only one encoding gene [9]. Members of the HMGA family are characterized by repeats of three amino acid sequences known as “AT hooks” that preferentially bind to AT-rich sequences in DNA. The binding of HMGA1 to DNA changes the DNA structure and regulates transcriptional complexes to regulate gene expression [10]. HMGA contains a small family of non-histone chromatin factor proteins that regulate gene transcription and can enhance or inhibit transcription factor activity [11].

Although the role of HMGA2 in the metastasis and growth of malignant tumors in the esophagus, stomach, and colorectum has been examined [9], the role of HMGA1 and its underlying mechanism remain poorly understood. Notably, overexpression of HMGA1 has been detected in breast cancer and neuroblastoma. HMGA1 binds to receptors for advanced glycation end-products and regulates the invasion and metastasis of triple-negative breast cancer cells. In neuroblastoma, HMGA1 is regulated by MYCN in the MYC protein family, and abnormal expression of MYCN affects overexpression of HMGA1 [12,13]. In patients with endometrioid endometrial carcinoma, high HMGA1 expression was associated with histological grade and tumor size as negative prognostic factors influenced by HMGA2 [14]. In addition, in patients with pancreatic cancer, HMGA1 promotes tumor development through the PI3-K/Akt cellular signaling pathway, and thus elevated HMGA1 expression is associated with poor prognosis [15]. Previous studies have reported a relationship between HMGA1/HMGA2 and gastric cancer. High levels of HMGA2 protein were significantly correlated with T stage, N stage, lymphatic infiltration, perineural infiltration, and TNM stage but were not confirmed to be related to survival [16]. In the present study, we evaluated the prognostic value of HMGA1 in gastric cancer and its relationship with cancer invasiveness.

## 2. Materials and Methods

### 2.1. Tissue Microarray Construction

This retrospective study of medical records was approved by the Institutional Review Board (IRB) of Cardinal Tien Hospital (IRB number: CTH-101-3-5-054) and the requirement for patient consent was waived. To construct the tissue microarray, we selected 181 patients for whom both tumor and adjacent non-tumor samples were available. Briefly, a tissue microarray was constructed as previously described. All tissue samples were immersed in formalin and embedded in paraffin. Selected tissue samples were sectioned on glass slides and stained with hematoxylin and eosin. After selecting the tumor and non-tumor samples, the area of interest in the paraffin block was perforated using a 2.0 mm cylindrical specimen, and the specimen was inserted into the recipient paraffin block to form a complete tissue array. Finally, 5 mm slices were cut from the complete array blocks and attached to glass slides, followed by histological and immunohistochemical staining.

### 2.2. Immunohistochemistry and Quantification

**Figure 1:**
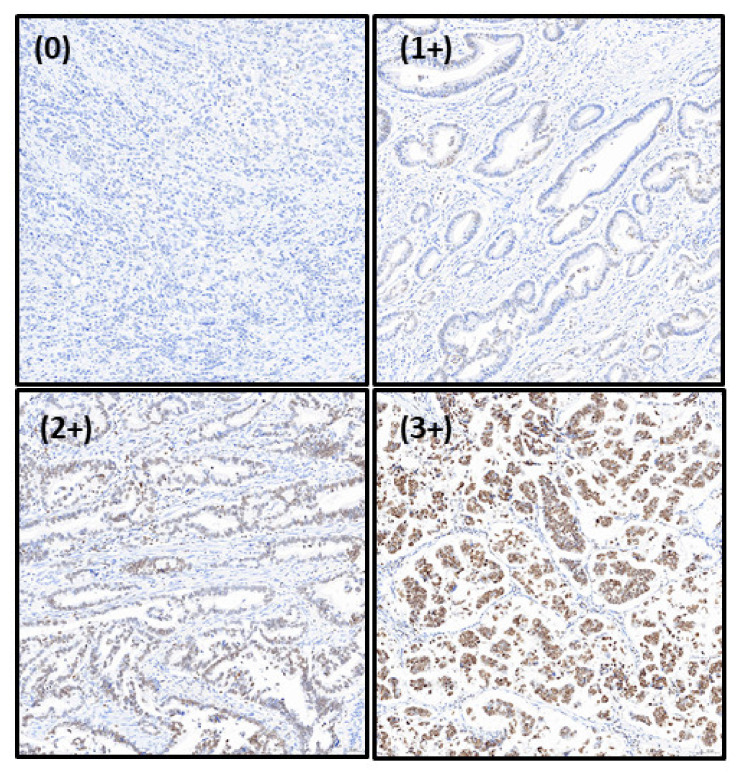
Representative images of different staining intensities for HMGA1 in gastric cancer. HMGA1, high mobility group A 1.

Briefly, the tissue sections were heated at 75 °C for 1 h, and then xylene and alcohol were added at different concentrations for deparaffinization and rehydration, respectively. The tissue sections were processed in citrate buffer (pH 6.0) for heat-induced antigen retrieval at 95 °C for 15 min. The prepared sections were blocked with normal goat serum and incubated with the primary antibody for 1 h at room temperature. After washing with phosphate-buffered saline, the corresponding secondary antibody was added and incubated at room temperature for 1 h. The slides were then washed with phosphate-buffered saline to remove the secondary antibody and horseradish peroxidase-conjugated secondary antibody was added, followed by incubation at room temperature for 30 min. Simultaneously, 0.01% 3,3-diaminobenzidine tetrahydrochloride (Sigma-Aldrich, St. Louis, MO, USA) was added to develop the immunostaining signal. The time was adjusted according to the color status and maintained under the same conditions for each tissue section. All immunohistochemical-stained slides were reviewed, and the immunostaining intensity was marked as 0 for no staining, 1 for weak staining, 2 for medium staining, and 3 for strong staining (Figure 1). The percentage of dyeing for each core, ranging from 0% to 100%, was also recorded. Finally, the H-scores were calculated from 0 to 300 by multiplying the staining intensity by the percentage of each score.

### 2.3. Primary Antibody

The following primary antibodies were used: HMGA1 (1:50, rabbit, 12094, Cell Signaling Technology, Danvers, MA, USA), Ki-67 (1:100, mouse, 350503, BioLegend, San Diego, CA, USA), cleaved caspase-3 (1:100, rabbit, 9664, Cell Signaling Technology), CD31 (1:500, rabbit, 250590, Abbiotec, Escondido, CA, USA), E-cadherin (1:100, rabbit, ab40772, Abcam, Cambridge, UK), N-cadherin (1:75, rabbit, ab76011, Abcam), fibronectin (1:50, mouse, SC-8422, Santa Cruz Biotechnology, Dallas, TX, USA), protein kinase B (AKT)-phosphorylated (1:50, mouse, GTX11901, GeneTex, Irvine, CA, USA), extracellular signal-regulated kinase (ERK)-phosphorylated (1:200, rabbit, AF1018 R&D Systems, Minneapolis, MN, USA), signal transducer and activator of transcription 3 (STAT3)-phosphorylated (1:50, rabbit, catalog number: ab 76315, Abcam), and activated protein kinase (AMPK)-phosphorylated (1:100, rabbit, 2535, Cell Signaling Technology).

### 2.4. Statistical Analysis

All statistical analyses were performed using SPSS software (version 20.0; SPSS, Inc., Chicago, IL, USA). To compare high and low HMGA1 expression levels, the chi-square test was used to analyze categorical variables. Bivariate correlation was used to determine the relationship between relevant tumorigenesis markers and HMGA1. For overall survival, the hazard ratio probability was calculated based on high or low HMGA1 expression. Finally, a Kaplan–Meier survival plot with the log-rank test was applied. All statistical tests were two-sided, and the results were considered statistically significant at *p* < 0.05.

## 3. Results

We evaluated samples from 181 patients with gastric cancer (122 females and 59 males) with an average age of 71.1 years. We further divided the patients by age as those over and under 65 years. Younger subjects exhibited higher HMGA1 expression (*p* = 0.015). Sex, degree of differentiation, and cancer stage did not affect HMGA1 expression (Table 1).

**Figure 2:**
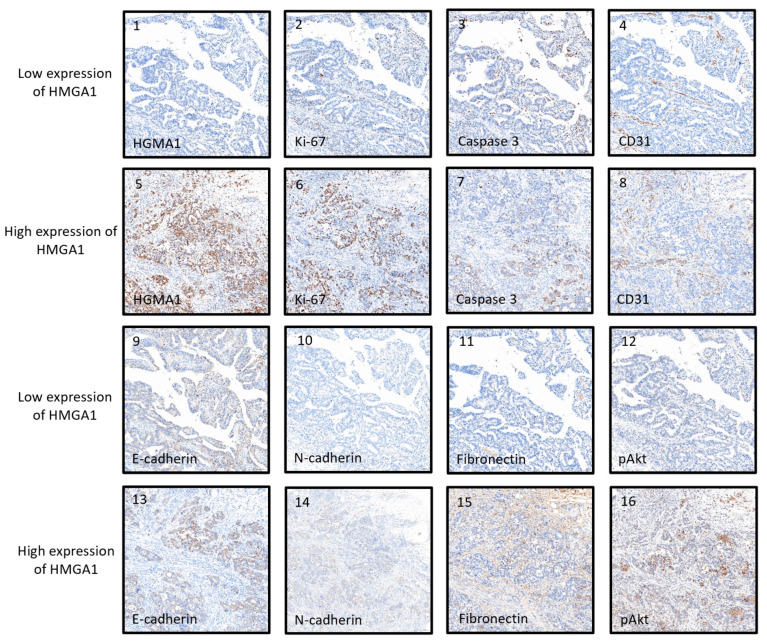
Representative images of HMGA1-associated tumorigenesis proteins. HMGA1, high mobility group A 1.

We used immunohistochemical staining to explore the relationship between HMGA1 expression and common tumorigenic proteins associated with proliferation (Ki-67), apoptosis (cleaved-caspase 3), angiogenesis (CD31), epithelial-mesenchymal transition (EMT; E-cadherin, N-cadherin, and fibronectin), and tyrosine kinase (pAkt, pErk, pSTAT3, and pAMPK). Pearson correlation analysis revealed that the *p*-values of Ki-67, caspase 3, CD31, E-cadherin, N-cadherin, fibronectin, pAkt, and pErk were <0.05, whereas pSTAT3 and pAMPK showed non-significant results (Table 2). Representative images of these proteins are shown in Figure 2.

**Figure 3:**
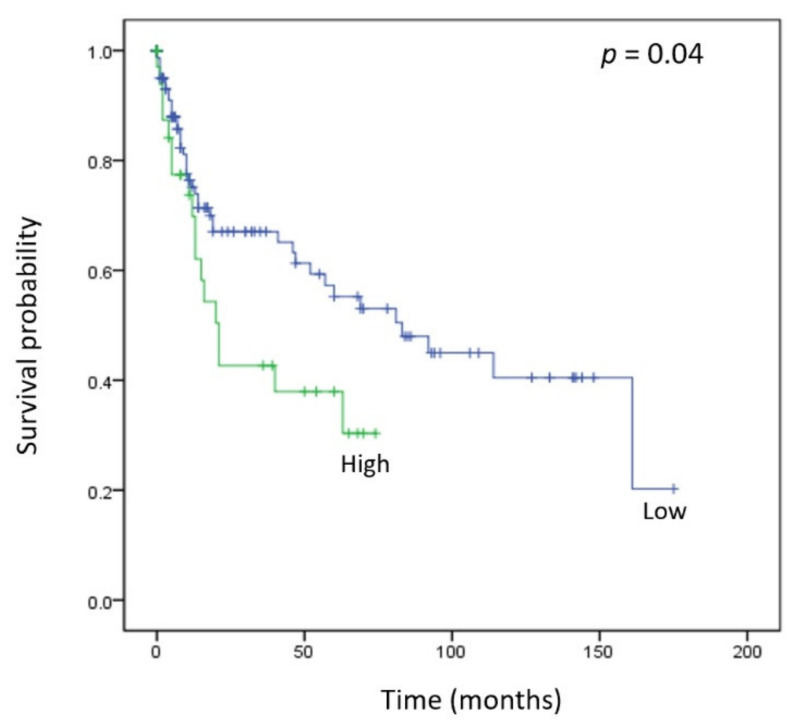
Kaplan–Meier plot of HMGA1. HMGA1, high mobility group A 1.

After identifying tumorigenic proteins associated with HMGA1, we assessed the prognostic value of HMGA1 in gastric cancer. In the Kaplan–Meier plot, higher expression levels of HMGA1 were correlated with relatively poor survival (*p* = 0.04) (Figure 3). Moreover, high HMGA1 expression presented a higher hazard ratio, which was 1.78-fold greater than that observed in samples with low HMGA1 expression. Additionally, the hazard ratio during the late stage was higher than that during the early stage (Table 3).

## 4. Discussion

We observed that high HMGA1 expression was associated with poor overall survival. Moreover, higher HMGA1expression was associated with a risk of harm, which was 1.78-fold greater than that observed in the low expression group. A previous in vitro study reported that HMGA1 regulates EMT and accelerates the development of gastric cancer [17]. Our tissue microarray results revealed similar findings, along with the involvement of additional proteins. We examined the underlying tumorigenic proteins and found that HMGA1 was associated with proliferation, apoptosis, angiogenesis, and EMT. Moreover, among the examined tyrosine kinases, we detected a negative correlation with pErk and a positive correlation with pAkt. These findings indicate that HMGA1 expression in gastric cancer is a marker of poor prognosis.

Ki-67 is a common protein and indicator of proliferation [18]. Previous studies showed that among malignant gliomas, HMGA1 is associated with Ki-67 [19]. High Ki-67 expression is an indicator of poor prognosis [20,21,22] and poor 5-year survival rate in patients with gastric cancer [23]. If the tumor proliferation rate is high, tumor apoptosis or necrosis is relatively elevated. During apoptosis, caspase-3 is among the main proteins involved in proteolytic degradation [24]. Based on experiments using rat and human thyroid cancer cells, high HMGA1 expression can stimulate the activation of caspase-3 and initiate cell apoptosis [25]. However, another report suggested that the expression of caspase-3 in patients with gastric cancer is related to better clinicopathological characteristics and a better prognosis after curative surgery [26]. Tumor cell proliferation requires a blood supply of nutrients; therefore, angiogenesis is another important factor correlated with tumor growth. CD31 is a marker of angiogenesis and cell migration, and its ability to resist cell death plays an important role in maintaining the stability of endothelial cells [27,28]. CD31 is often employed as an indicator of the extent of tumor invasion in cancer [29]. In gastric cancer, CD31 can be used as an indicator of survival related to angioinvasion [30]. In contrast to non-tumor conditions, higher HMGA1 expression has been correlated with lower expression of CD31 in a mouse model of pulmonary hypertension [31]. This finding supports the correlation between angiogenesis or blood vessel formation and HMGA1, with distinct results in tumor and non-tumor conditions.

Epithelial cells differentiate into active mesenchymal cells via a process known as EMT, which promotes tissue fibrosis and tumor progression [32,33,34]. EMT is related to carcinogenesis and enhanced the mobility, invasiveness, and anti-apoptotic properties of tumor cells [35]. Inhibition of HMGA1 can regulate EMT and affect the metastasis and prognosis of cancer cells, which has been reported in non-small cell lung cancer, cervical cancer, breast cancer, thyroid cancer, and gastric cancer [17,36,37,38,39]. Based on our findings, HMGA1 was significantly and positively associated with N-cadherin and fibronectin in gastric cancer, contributing to tumor invasiveness and ultimately indicating a poor prognosis.

Activation of Akt and Erk is often abnormally regulated in cancer; these proteins play important roles in the growth and survival of tumor cells [40,41]. In studies of kidney, liver, breast, and lung cancers, inhibition of HMGA1 reportedly the performance of pAKT and inhibited tumor growth and metastasis [42,43,44,45]. In addition, breast cancer and neuroblastoma cell line experiments showed that inhibition of HMGA1 reduces the performance of pErk, affecting tumor cell proliferation, migration, and invasion, and increasing apoptosis [46,47]. Our results showed a positive correlation between HMGA1 and pAkt, which is consistent with the results of previous studies. However, we detected a negative correlation between HMGA1 and pErk, with a borderline significant *p*-value of 0.048. Further studies are needed to confirm the relationship between pErk and HMGA1 expression.

Our study has several limitations. We identified several proteins related to the expression of HMGA1; however, an in-depth assessment of the relationship between these proteins and HMGA1 was not performed. Our findings are similar to those of observational studies and not mechanistic studies. However, analysis of a larger number of human specimens may provide further evidence for the role of HMGA1 in gastric cancer.

## 5. Conclusions

High HMGA1 expression indicates a poor prognosis for gastric cancer. Moreover, the expression of HMGA1 can affect tumor proliferation, apoptosis, angiogenesis, and N-cadherin and fibronectin expression, possibly through pAkt or pERK. However, additional studies of the underlying mechanisms are needed.

## Figures and Tables

**Table 1:** Demographic data of patients with gastric cancer and HMGA1 expression.

Factors		HMGA1 Low Expression	HMGA1 High Expression	*p*-Value
Age (years)	<65	38 (76.0%)	12 (24.0%)	0.015
	≥65	109 (83.2)	22 (16.8%)	
Gender	Female	99 (81.1%)	23 (18.9%)	0.973
	Male	48 (81.4%)	11 (18.6%)	
Differentiation	Well	44 (84.6%)	8 (15.4%)	0.460
	Moderate	103 (79.8%)	26 (20.2%)	
Stage	I and II	60 (81.1%)	14 (18.9%)	0.970
	III and IV	87 (81.3%)	20 (18.7%)	

HMGA1, high mobility group A 1.

**Table 2:** Correlation of HMGA1 with invasiveness markers and tyrosine kinase.

Biomarkers		r	*p*-Value
Proliferation	Ki-67	0.402	<0.001
Apoptosis	Caspase 3	0.193	<0.001
Angiogenesis	CD31	0.167	0.002
EMT	E-cadherin	0.176	0.001
	N-cadherin	0.154	0.005
	Fibronectin	0.340	<0.001
Tyrosine kinase	pAkt	0.340	<0.001
	pErk	−0.109	0.048
	pSTAT3	0.003	0.950
	pAMPK	0.022	0.691

EMT, epithelial-mesenchymal transition; HMGA1, high-mobility group A 1; pAKT, phosphorylated protein kinase B; pAMPK, phosphorylated-activated protein kinase; pERK, phosphorylated-extracellular signal-regulated kinase; pSTAT3, phosphorylated signal transducer and activator of transcription 3.

**Table 3:** Common classification and clinical stage hazard ratio of the HMGA1.

		Hazard Ratio (95% CI)	*p*-Value
HMGA1 expression	Low	Reference	
	High	1.78 (1.01–3.14)	0.045
Age (years)	<65	Reference	
	>=65	0.70 (0.41–1.18)	0.184
Gender	Female	Reference	
	Male	0.74 (0.42–1.32)	0.311
Differentiation	Moderate	Reference	
	Well	1.74 (0.94–3.34)	0.079
Stage	I and II	Reference	
	III and IV	2.50 (1.44–4.35)	0.001

## Data Availability

The data presented in this study are available upon request from the corresponding author. The data are not publicly available because they still need to be protected, so they are not provided.

## References

[1] Jiang Y., Yu Y. (2017). Transgenic and gene knockout mice in gastric cancer research. Oncotarget.

[2] Poh A.R., O’Donoghue R.J., Ernst M., Putoczki T.L. (2016). Mouse models for gastric cancer: Matching models to biological questions. J. Gastroenterol. Hepatol..

[3] Thrift A.P., El-Serag H.B. (2020). Burden of gastric cancer. Clin. Gastroenterol. Hepatol..

[4] Digklia A., Wagner A.D. (2016). Advanced gastric cancer: Current treatment landscape and future perspectives. World J. Gastroenterol..

[5] Song Z., Wu Y., Yang J., Yang D., Fang X. (2017). Progress in the treatment of advanced gastric cancer. Tumour Biol..

[6] Tan Z. (2019). Recent advances in the surgical treatment of advanced gastric cancer: A review. Med. Sci. Monit..

[7] Garattini S.K., Basile D., Cattaneo M., Fanotto V., Ongaro E., Bonotto M., Negri F.V., Berenato R., Ermacora P., Cardellino G.G. (2017). Molecular classifications of gastric cancers: Novel insights and possible future applications. World J. Gastrointest. Oncol..

[8] Fassan M., Saraggi D., Balsamo L., Cascione L., Castoro C., Coati I., De Bernard M., Farinati F., Guzzardo V., Valeri N. (2016). Let-7c down-regulation in Helicobacter pylori-related gastric carcinogenesis. Oncotarget.

[9] Meireles Da Costa N., Ribeiro Pinto L.F., Nasciutti L.E., Palumbo A. (2019). The prominent role of HMGA proteins in the early management of gastrointestinal cancers. BioMed Res. Int..

[10] Cui T., Leng F. (2007). Specific recognition of AT-rich DNA sequences by the mammalian high mobility group protein AT-hook 2: A SELEX study. Biochemistry.

[11] Shah S.N., Resar L.M. (2012). High mobility group A1 and cancer: Potential biomarker and therapeutic target. Histol. Histopathol..

[12] Giannini G., Cerignoli F., Mellone M., Massimi I., Ambrosi C., Rinaldi C., Gulino A. (2005). Molecular mechanism of HMGA1 deregulation in human neuroblastoma. Cancer Lett..

[13] Mendez O., Perez J., Soberino J., Racca F., Cortes J., Villanueva J. (2019). Clinical implications of extracellular HMGA1 in breast cancer. Int. J. Mol. Sci..

[14] Palumbo A., de Sousa V.P.L., Esposito F., De Martino M., Forzati F., Moreira F.C.B., Simão T.D.A., Nasciutti L.E., Fusco A., Pinto L.F.R. (2019). Overexpression of HMGA1 figures as a potential prognostic factor in endometrioid endometrial carcinoma (EEC). Genes.

[15] Liau S.S., Rocha F., Matros E., Redston M., Whang E. (2008). High mobility group AT-hook 1 (HMGA1) is an independent prognostic factor and novel therapeutic target in pancreatic adenocarcinoma. Cancer.

[16] Jun K.H., Jung J.H., Choi H.J., Shin E.Y., Chin H.M. (2015). HMGA1/HMGA2 protein expression and prognostic implications in gastric cancer. Int. J. Surg..

[17] Jin G.H., Shi Y., Tian Y., Cao T.T., Mao Y., Tang T.Y. (2020). HMGA1 accelerates the malignant progression of gastric cancer through stimulating EMT. Eur. Rev. Med. Pharmacol. Sci..

[18] Menon S.S., Guruvayoorappan C., Sakthivel K.M., Rasmi R.R. (2019). Ki-67 protein as a tumour proliferation marker. Clin. Chim. Acta.

[19] Pang B., Fan H., Zhang I.Y., Liu B., Feng B., Meng L., Zhang R., Sadeghi S., Guo H., Pang Q. (2012). HMGA1 expression in human gliomas and its correlation with tumor proliferation, invasion and angiogenesis. J. Neurooncol..

[20] Kloppel G., La Rosa S. (2018). Correction to: Ki67 labeling index: Assessment and prognostic role in gastroenteropancreatic neuroendocrine neoplasms. Virchows Arch..

[21] Luo G., Hu Y., Zhang Z., Wang P., Luo Z., Lin J., Cheng C., Yang Y. (2017). Clinicopathologic significance and prognostic value of Ki-67 expression in patients with gastric cancer: A meta-analysis. Oncotarget.

[22] Yilmaz H., Demirag G., Sullu Y., Yilmaz A. (2021). Predictive significance of Ki-67 and platelet lymphocyte ratio in patients with gastric cancer receiving neoadjuvant FLOT chemotherapy. J. Coll. Physicians Surg. Pak..

[23] Xie J., Zhao Y., Zhou Y., He Q., Hao H., Qiu X., Zhao G., Xu Y., Xue F., Chen J. (2021). Predictive value of combined preoperative carcinoembryonic antigen level and Ki-67 index in patients with gastric neuroendocrine carcinoma after radical surgery. Front. Oncol..

[24] Mansoori B., Mohammadi A., Shirjang S., Baradaran B. (2016). HMGI-C suppressing induces P53/caspase9 axis to regulate apoptosis in breast adenocarcinoma cells. Cell Cycle.

[25] Fedele M., Pierantoni G.M., Berlingieri M.T., Battista S., Baldassarre G., Munshi N., Dentice M., Thanos D., Santoro M., Viglietto G. (2001). Overexpression of proteins HMGA1 induces cell cycle deregulation and apoptosis in normal rat thyroid cells. Cancer Res..

[26] Huang K.-H., Fang W.-L., Li A.F.-Y., Liang P.-H., Wu C.-W., Shyr Y.-M., Yang M.-H. (2018). Caspase-3, a key apoptotic protein, as a prognostic marker in gastric cancer after curative surgery. Int. J. Surg..

[27] Lertkiatmongkol P., Liao D., Mei H., Hu Y., Newman P.J. (2016). Endothelial functions of platelet/endothelial cell adhesion molecule-1 (CD31). Curr. Opin. Hematol..

[28] Winn R.K., Harlan J.M. (2005). The role of endothelial cell apoptosis in inflammatory and immune diseases. J. Thromb. Haemost..

[29] Ogut B., Ekinci O., Celik B., Unal E.R., Dursun A. (2020). Comparison of the efficiency of transgelin, smooth muscle myosin, and CD31 antibodies for the assessment of vascular tumor invasion and free tumor deposits in gastric, pancreatic, and colorectal adenocarcinomas. Indian J. Pathol. Microbiol..

[30] Li Y., Guo X.B., Wei Y.H., Kang X.L. (2021). Serum CXCL13 and PECAM-1 can be used as diagnostic and prognostic markers in elderly patients with gastric cancer. Clin. Transl. Oncol..

[31] Hopper R.K., Moonen J.R., Diebold I., Cao A., Rhodes C.J., Tojais N.F., Hennigs J.K., Gu M., Wang L., Rabinovitch M. (2016). In pulmonary arterial hypertension, reduced BMPR2 promotes endothelial-to-mesenchymal transition via HMGA1 and its target Slug. Circulation.

[32] Gonzalez D.M., Medici D. (2014). Signaling mechanisms of the epithelial-mesenchymal transition. Sci. Signal..

[33] Lamouille S., Xu J., Derynck R. (2014). Molecular mechanisms of epithelial-mesenchymal transition. Nat. Rev. Mol. Cell Biol..

[34] Zhang Y., Weinberg R.A. (2018). Epithelial-to-mesenchymal transition in cancer: Complexity and opportunities. Front. Med..

[35] Mittal V. (2018). Epithelial mesenchymal transition in tumor metastasis. Annu. Rev. Pathol..

[36] Huang R., Huang D., Dai W., Yang F. (2015). Overexpression of HMGA1 correlates with the malignant status and prognosis of breast cancer. Mol. Cell. Biochem..

[37] Ma Y., Li X., Chen S., Du B., Li Y. (2019). MicroRNA-4458 suppresses migration and epithelial-mesenchymal transition via targeting HMGA1 in non-small-cell lung cancer cells. Cancer Manag. Res..

[38] Xie J., Chen Q., Zhou P., Fan W. (2021). Circular RNA hsa_circ_0000511 improves epithelial mesenchymal transition of cervical cancer by regulating hsa-mir-296-5p/HMGA1. J. Immunol. Res..

[39] Zhong J., Liu C., Zhang Q.-H., Chen L., Shen Y.-Y., Chen Y.-J., Zeng X., Zu X.-Y., Cao R.-X. (2017). TGF-beta1 induces HMGA1 expression: The role of HMGA1 in thyroid cancer proliferation and invasion. Int. J. Oncol..

[40] Olea-Flores M., Zuniga-Eulogio M.D., Mendoza-Catalan M.A., Rodriguez-Ruiz H.A., Castaneda-Saucedo E., Ortuno-Pineda C., Padilla-Benavides T., Navarro-Tito N. (2019). Extracellular-signal regulated kinase: A central molecule driving epithelial-mesenchymal transition in cancer. Int. J. Mol. Sci..

[41] Vincent E.E., Elder D.J., Thomas E.C., Phillips L., Morgan C., Pawade J., Sohail M., May M.T., Hetzel M.R., Tavaré J.M. (2011). Akt phosphorylation on Thr308 but not on Ser473 correlates with Akt protein kinase activity in human non-small cell lung cancer. Br. J. Cancer.

[42] Chen X.-G., Liu F., Song X.-F., Wang Z.-H., Dong Z.-Q., Hu Z.-Q., Lan R.-Z., Guan W., Zhou T.-G., Xu X.-M. (2010). Rapamycin regulates Akt and ERK phosphorylation through mTORC1 and mTORC2 signaling pathways. Mol. Carcinog..

[43] Liu L., Zhang S., Hu L., Liu L., Guo W., Zhang J. (2017). HMGA1 participates in MHCC97H cell proliferation and invasion through the ILK/Akt/GSK3beta signaling pathway. Mol. Med. Rep..

[44] Takaha N., Sowa Y., Takeuchi I., Hongo F., Kawauchi A., Miki T. (2012). Expression and role of HMGA1 in renal cell carcinoma. J. Urol..

[45] Zhang Y., Ma T., Yang S., Xia M., Xu J., An H., Yang Y., Li S. (2011). High-mobility group A1 proteins enhance the expression of the oncogenic miR-222 in lung cancer cells. Mol. Cell. Biochem..

[46] Hanna R., Abdallah J., Abou-Antoun T. (2020). A novel mechanism of 17-AAG therapeutic efficacy on HSP90 inhibition in MYCN-amplified neuroblastoma cells. Front. Oncol..

[47] Mendez O., Peg V., Salvans C., Pujals M., Fernandez Y., Abasolo I., Pérez J., Matres A., Valeri M., Gregori J. (2018). Extracellular HMGA1 promotes tumor invasion and metastasis in triple-negative breast cancer. Clin. Cancer Res..

